# Association of LDL-C level with neoatherosclerosis and plaque vulnerability in patients with late restenosis: an optical coherence tomography study

**DOI:** 10.1007/s10554-023-02956-1

**Published:** 2023-10-07

**Authors:** Zhijiang Liu, Chancui Deng, Ranzun Zhao, Guanxue Xu, Zhixun Bai, Zhenglong Wang, Wei Zhang, Yi Ma, Xingwei Hu, Caide Jin, Panke Chen, Shuai Ma, Bei Shi

**Affiliations:** 1 Department of Cardiology, Affiliated Hospital of Zunyi Medical University, Zunyi, 563000 China; 2 Department of Cardiology, the Fifth Affiliated Hospital of Zunyi Medical University, Zhuhai, China

**Keywords:** Optical coherence tomography, In-stent restenosis, Neoatherosclerosis, Thin-cap fibroatheroma, Low-density lipoprotein cholesterol

## Abstract

Neoatherosclerosis (NA) is a significant contributor to late stent failure; however, predictors of late in-stent restenosis (ISR) with NA have not been systematically reported. This study aimed to identify predictors of NA incidence and plaque vulnerability in patients with late ISR and the role of low-density lipoprotein cholesterol (LDL-C) levels in this process. A total of 216 patients with 216 lesions who underwent optical coherence tomography (OCT) before interventional procedure for late drug-eluting stent ISR were enrolled and divided into NA and non-NA groups based on OCT findings. Results showed that higher LDL-C levels were associated with NA, thin-cap fibroatheroma (TCFA), intimal disruption, plaque erosion, and thrombosis. Multivariate regression analysis revealed that the LDL-C level was an independent risk factor for NA and TCFA. The LDL-C levels exhibited a significant predictive value for NA and TCFA, surpassing other factors such as stent age and other lipid types. In conclusion, a high LDL-C level is an independent predictor of NA incidence and plaque vulnerability in patients with late ISR.

**Supplementary Information:**

The online version contains supplementary material available at 10.1007/s10554-023-02956-1.

## Introduction

Despite the ongoing evolution and iterations of drug-eluting stent (DES) technologies, in-stent restenosis (ISR) remains a major challenge in the current interventional field. With the emergence and wide application of endovascular imaging technology, increasing evidence has shown that neoatherosclerosis (NA) is a significant contributor to late stent failure and the promotion of major adverse cardiovascular events (MACEs) [1–4]. However, the mechanism of NA formation is complex and remains unclear. Previous studies have revealed that the occurrence of NA is related to factors such as stent implantation time, stent type, current smoking status, chronic kidney disease (CKD), and the use of angiotensin-converting enzyme inhibitor/angiotensin receptor blocker (ACEI/ARB) [1, 5, 6]. However, it is worth noting that low-density lipoprotein cholesterol (LDL-C) plays an important role in the occurrence and progression of atherosclerosis. Studies of in situ lesions have shown that intensive lipid-lowering therapy can stabilize and reverse plaques [7, 8], but its role in NA formation remains controversial. Some studies have shown that elevated LDL-C levels facilitate NA formation [9–11], whereas others have shown that NA formation is not related to LDL-C levels [1, 12, 13]. More importantly, no systematic studies on the risk factors for late ISR with NA formation have been reported, and it remains unclear whether elevated LDL-C levels are also involved in the progression of late NA. In addition, optical coherence tomography (OCT) has become the preferred intravascular imaging method for identifying NA and vulnerable plaques in vivo [14, 15]. Therefore, this study used OCT to systematically investigate the relationship between LDL-C level and NA and plaque vulnerability in late ISR lesions.

## Methods

### Study population

**Fig. 1:**
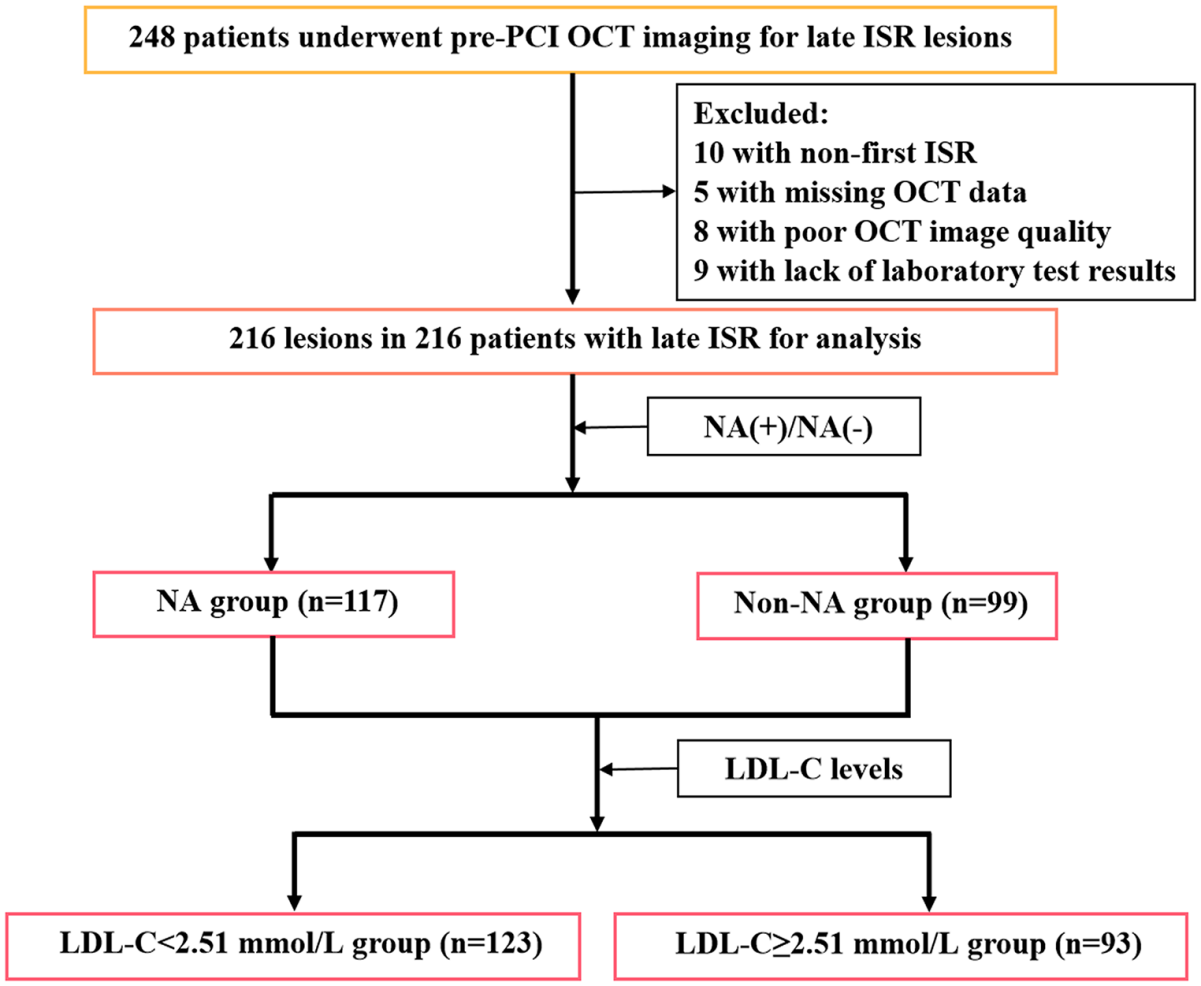
Study flow diagram. ISR: in-stent restenosis; LDL-C: low-density lipoprotein cholesterol; NA: neoatherosclerosis; OCT: optical coherence tomography; PCI: percutaneous coronary intervention

Patients with late ISR (stent age > 1 year) confirmed by coronary angiography (CAG) who underwent OCT imaging at our institution between March 2015 and January 2023 were retrospectively enrolled. ISR was defined as a percent diameter stenosis of > 50% within the stent segment based on a previous report [16]. Patients with inadequate laboratory test results, poor OCT image quality, and non-first ISR (two or more ISRs in the same lesion) and those who underwent any interventional procedure before OCT imaging were excluded. Notably, if two ISR lesions were attributed to the same patient, only the ISR lesion with the most severe stenosis was included. Finally, 216 patients with 216 ISR lesions were included in the study (Fig. 1). Two distinct scenarios warranted follow-up CAG in this study: routine surveillance after the treatment of other lesions and evidence of myocardial ischemia [17]. The study was conducted in accordance with the Declaration of Helsinki and approved by the ethics committee of our institution. All patients provided informed consent to participate in this study.

### Data collection and angiographic analysis

Data collection and angiographic analysis are described in Online Resource 1.

### OCT image acquisition and analysis

OCT image acquisition is described in Online Resource 1. All images were analyzed offline by LightLab OCT (Light Lab Imaging Inc., Westford, MA, USA). All cross-sectional images were preliminarily assessed qualitatively and excluded from the analysis if any part of the stent was outside the field of view or if the image quality was poor. Qualitative analysis was performed for each frame, and quantitative analysis was performed for the minimum lumen area (MLA) site. OCT analysis was performed by two investigators who were blinded to the clinical data of the patients. In case of disagreement between the two investigators, a third analyst read the images independently and reached a consensus. NA was defined as the observation of lipid or calcified neointimal formation on at least three consecutive cross-sectional OCT images [18]. A lipid neointima was defined as an area with a diffuse boundary of the intima with a hyposignal area of marked attenuation [19]. A calcified neointima was defined as a well-defined area with poor signal intensity [20]. Some ISR lesions contain both lipid and calcified neointima. Thin-cap fibroatheroma (TCFA) was defined as a lipid plaque with the thinnest fibrous cap thickness of < 65 μm and a lipid arc of > 90° [21]. Other definitions are provided in Online Resource 1.

### Statistical analysis

Data analysis is described in Online Resource 1.

## Results

### Baseline characteristics and angiographic characteristics

A total of 216 patients with 216 late ISR lesions were enrolled in this study, with a median age of 64 years; 79.6% were men. Based on OCT follow-up characteristics, the patients in this study were divided into the NA group (n = 117) and non-NA group (n = 99). The estimated glomerular filtration rate (eGFR) was lower, the stent age was longer, and the LDL-C, triglyceride (TG), total cholesterol (TC), apolipoprotein B (APOB), APOB/A1 ratio, and the proportion of patients with acute coronary syndrome (ACS) at follow-up were higher in the NA group (*p* < 0.05). There were no significant differences in other baseline characteristics and angiographic characteristics between the two groups (*p >* 0.05) (Tables 1 and 2).

**Table 1:** Baseline characteristics

		Overall (n = 216)	NA (n = 117)	Non-NA (n = 99)	*p*-value
**General information**					
	Age, year	64.00 (56.00–71.00)	65.00 (56.50–71.00)	63.00 (55.00–70.00)	0.356
	Male	172 (79.6)	93 (79.5)	79 (79.8)	0.955
	Current smoker	74 (34.3)	42 (35.9)	32 (32.3)	0.581
	Hypertension	128 (59.3)	67 (57.3)	61 (61.6)	0.517
	Diabetes mellitus	68 (31.5)	34 (29.1)	34 (34.3)	0.405
	LVEF, %	56.00 (49.25-61.00)	56.00 (46.00–59.00)	57.00 (50.00–61.00)	0.077
	Creatinine, µmol/L	84.00 (70.00-100.00)	85.00 (70.50–101.00)	83.00 (69.00–99.00)	0.179
	eGFR, mL/min/1.73 m^2^	107.74 ± 33.85	103.48 ± 32.96	112.76 ± 34.37	0.044
	eGFR < 60, mL/min/1.73 m^2^	15 (6.9)	12 (10.3)	3 (3.0)	0.037
	Fasting blood glucose, mmol/L	5.75 (4.91–6.77)	5.78 (4.89–6.75)	5.70 (4.98–6.77)	0.566
	TG, mmol/L	1.69 (1.24–2.56)	1.92 (1.30–2.79)	1.55 (1.19–2.37)	0.039
	TC, mmol/L	3.88 (3.42–4.83)	4.13 (3.3.55–5.28)	3.79 (3.21–4.41)	0.004
	HDL-C, mmol/L	1.11 (0.94–1.25)	1.05 (0.93–1.24)	1.15 (0.95–1.29)	0.054
	LDL-C, mmol/L	2.38 (1.98–2.89)	2.56 (2.08–3.13)	2.17 (1.72–2.71)	< 0.001
	APOA1, g/L	1.20 (1.07–1.33)	1.18 (1.06–1.32)	1.22 (1.11–1.34)	0.401
	APOB, g/L	0.73 (0.60–0.90)	0.78 (0.62–0.97)	0.67 (0.57–0.84)	0.003
	APOB/A1	0.61 (0.49–0.77)	0.65 (0.53–0.89)	0.55 (0.47–0.69)	0.003
	Stent age, months	48.00 (25.25-84.00)	60 (31.00–96.00)	48.00 (24.00–72.00)	0.023
**Clinical presentation**					
	ACS at stenting	85 (39.4)	50 (42.7)	35 (35.4)	0.269
	ACS at restenosis	63 (29.2)	48 (41.0)	15 (15.2)	< 0.001
**Medication at restenosis**					
	Aspirin	176 (81.5)	92 (78.6)	84 (84.8)	0.241
	Statin	183 (84.7)	97 (82.9)	86 (86.9)	0.420
	ACEI/ARB	176 (81.5)	90 (76.9)	86 (86.9)	0.061
	β-blocker	179 (82.9)	96 (82.1)	83 (83.8)	0.728
	Insulin	12 (5.6)	5 (4.3)	7 (7.1)	0.371

Data are expressed as the median (interquartile range), mean ± SD, or n (%). ACEI: angiotensin-converting enzyme inhibitor; ACS: acute coronary syndrome; APO: apolipoprotein; ARB: angiotensin receptor blocker; eGFR, estimated glomerular filtration rate; HDL-C: high-density lipoprotein cholesterol; LDL-C: low-density lipoprotein cholesterol; LVEF: left ventricular ejection fraction; NA: neoatherosclerosis; TC: total cholesterol; TG: triglyceride

**Table 2:** Angiographic characteristics

		Overall (n = 216)	NA (n = 117)	Non-NA (n = 99)	*p*-value
Lesion length, mm		12.05 (8.80-17.15)	12.80 (8.80-17.45)	11.20 (8.80–16.70)	0.348
Multivessel disease		170 (78.7)	97 (82.9)	73 (73.7)	0.101
Reference vessel diameter, mm		3.19 ± 0.41	3.20 ± 0.42	3.18 ± 0.40	0.804
MLD, mm		1.16 ± 0.22	1.14 ± 0.23	1.17 ± 0.20	0.293
Diameter stenosis, %		63.63 ± 6.00	64.14 ± 6.34	63.02 ± 5.55	0.173
ISR location					0.009
	LAD	133 (61.6)	73 (62.4)	60 (60.6)	
	LCX	23 (10.6)	6 (5.1)	17 (17.2)	
	RCA	60 (27.8)	38 (32.5)	22 (22.2)	
ISR pattern					0.317
	Focal	86 (39.8)	43 (36.8)	43 (43.4)	
	Diffuse	130 (60.2)	74 (63.2)	56 (56.6)	
Previous stent type					0.228
	First-generation DES	159 (73.6)	88 (75.2)	71 (71.7)	
	New-generation DES	46 (21.3)	21 (17.9)	25 (25.3)	
	Unknown	11 (5.1)	8 (6.8)	3 (3.0)	

Data are expressed as the median (interquartile range), mean ± SD, or n (%). DES: drug eluting stent; ISR: in-stent restenosis; LAD: left anterior descending artery; LCX: left circumflex artery; MLD: minimal lumen diameter; NA: neoatherosclerosis; RCA: right coronary artery

### OCT analysis of late ISR lesions

Detailed qualitative and quantitative OCT data are presented in Table 3. Similarities were found in the quantitative data between the two groups (*p* > 0.05). Qualitative analysis of the entire stent revealed that TCFA and intimal ruptures were found only in the NA group. Moreover, plaque erosion, neovascularization (NV), and thrombosis were significantly higher in the NA group (*p* < 0.01). The results of the qualitative analysis of the MLA site were similar to those of the entire stent analysis. TCFA, intimal ruptures, and thrombosis were only observed in the NA group. Although heterogeneous intima was predominant in both groups, the incidence of heterogeneous intima was significantly higher in the NA group compared with that in the non-NA group (*p* < 0.001).

**Table 3:** Optical coherence tomography characteristics

			Overall (n = 216)	NA (n = 117)	Non-NA (n = 99)	*p*-value
**Quantitative analysis**						
	MLA, mm^2^		1.68 ± 0.58	1.61 ± 0.63	1.76 ± 0.52	0.067
	Stent area (MLA site), mm^2^		6.58 ± 1.62	6.50 ± 1.58	6.68 ± 1.67	0.435
	Neointimal tissue area (MLA site), mm ^2^		4.90 ± 1.47	4.89 ± 1.45	4.92 ± 1.49	0.881
	Burden of neointimal tissue (MLA site), %		73.81 ± 8.73	74.61 ± 9.48	72.86 ± 7.70	0.136
**Qualitative analysis**						
	Neointima characteristics at entire stent					
		NA with lipid	112 (51.9)	112 (95.7)	0 (0.0)	< 0.001
		NA with calcium	40 (18.5)	40 (34.2)	0 (0.0)	< 0.001
		TCFA	42 (19.4)	42 (35.9)	0 (0.0)	< 0.001
		Intimal disruption	40 (18.5)	40 (34.2)	0 (0.0)	< 0.001
		Plaque erosion	60 (27.8)	52 (44.4)	8 (8.1)	< 0.001
		NV	96 (44.4)	62 (53.0)	34 (34.3)	0.006
		Thrombus	54 (25.0)	46 (39.3)	8 (8.1)	< 0.001
	Neointima characteristics at MLA site					
		Homogeneous	44 (20.4)	8 (6.8)	36 (36.4)	< 0.001
		Heterogeneous	172 (79.6)	109 (93.2)	63 (63.6)	< 0.001
		TCFA	28 (13.0)	28 (23.9)	0 (0.0)	< 0.001
		Intimal disruption	25 (11.6)	25 (21.4)	0 (0.0)	< 0.001
		Thrombus	27 (12.5)	27 (23.1)	0 (0.0)	< 0.001

Data are expressed as the median (interquartile range) or n (%). MLA: minimum lumen area; NA: neoatherosclerosis; NV: neovascularization; TCFA: thin-cap fibroatheroma

### Prediction of NA

Univariate regression analysis showed that blood creatinine levels, eGFR, stent age, TG, TC, LDL-C, APOB, and APOB/A1 ratio were associated with NA formation (*p* < 0.05). Multivariate regression analysis revealed that LDL-C and TG levels and stent age were associated with NA formation (*p* < 0.05) (Online Resource 2). To avoid interactions between lipid types in the multifactor analysis, confounding factors were adjusted for lipid types with differences in the univariate regression analysis to evaluate their roles in NA formation. Model 1 was corrected for age and sex. Model 2 was based on Model 1 with the addition of current smoking status, hypertension, diabetes mellitus, stent age, ACEI/ARB, and first-generation DES. Model 3 was based on Model 2 with the addition of left ventricular ejection fraction, fasting blood glucose, creatinine, eGFR, and eGFR < 60mL/min/1.73 m^2^. According to the regression analysis results of the different models, lipid profiles play an important role in NA formation. TG, TC, LDL-C, APOB, and APOB/A1 ratio were still related to NA formation after multifactor correction (*p* < 0.05) (Online Resource 3). In addition, stent age was still correlated with NA formation after correction for different lipid types (*p* < 0.05) (Online Resource 4).

### Receiver operating characteristic analysis

**Fig. 2:**
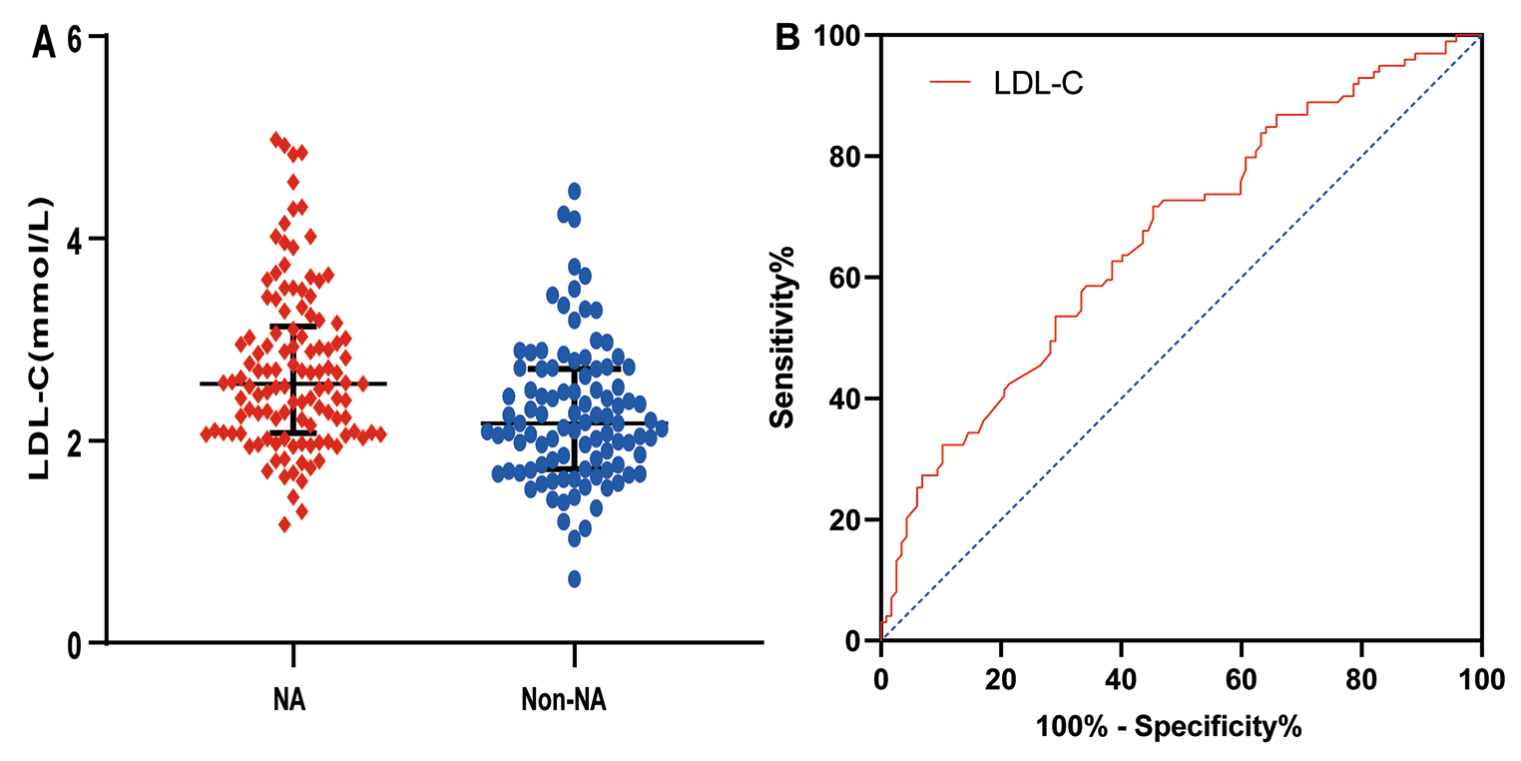
LDL-C level and ROC curve analysis for NA. LDL-C: low-density lipoprotein cholesterol; NA: neoatherosclerosis; ROC: receiver operating characteristic

The level of LDL-C in the NA group was higher than that in the non-NA group (*p* < 0.001) (Fig. 2-A). Therefore, the predictive value of LDL-C for NA was further evaluated using a receiver operating characteristic (ROC) curve, and the area under the curve (AUC) was 0.665 (95% confidence interval [CI], 0.593–0.737; *p* < 0.001) (Fig. 2-B). Moreover, we evaluated the AUCs of APOB/A1 ratio, APOB, TG, TC, and stent age, which were 0.617 (95% CI, 0.542–0.692; *p* = 0.003), 0.616 (95% CI, 0.541–0.690; *p* = 0.004), 0.582 (95% CI, 0.505–0.658; *p* = 0.039), 0.613 (95% CI, 0.538–0.688; *p* = 0.0042), and 0.590 (95% CI, 0.513–0.666; *p* = 0.0235), respectively (Online Resource 7).

### Subgroup analyses

#### The relationship between LDL-C levels and plaque vulnerability

**Fig. 3:**
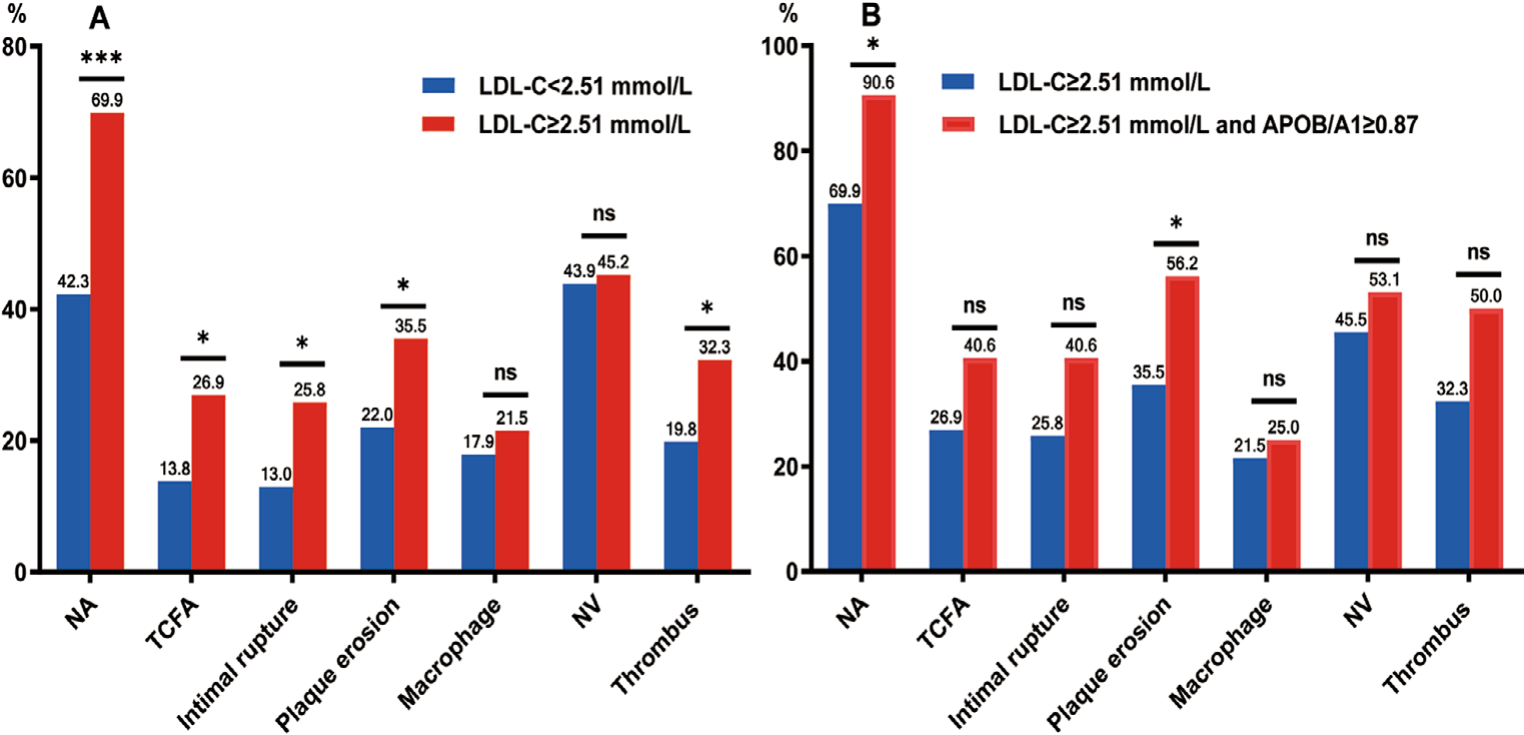
Comparison of plaque characteristics between different groups. APO: apolipoprotein; LDL-C: low-density lipoprotein cholesterol; NA: neoatherosclerosis; NV: neovascularization; TCFA: thin-cap fibroatheroma. ***p < 0.001; *p < 0.05

To further evaluate the relationship between LDL-C levels and plaque vulnerability in patients with late ISR, we divided subjects into two groups based on the cut-off values of LDL-C as follows: LDL-C < 2.51 mmol/L (n = 123) and LDL-C ≥ 2.51 mmol/L (n = 93). The clinical, CAG, and OCT data of the patients are presented in Tables 4 and 5. TG, TC, APOB, and APOB/A1 ratio and the incidences of NA, TCFA, intimal rupture, plaque erosion, and thrombus in the LDL-C ≥ 2.51 mmol/L group were higher than those in the LDL-C < 2.51 mmol/L group (*p* < 0.05) (Fig. 3-A). Similarities were found in other clinical data, CAG findings, OCT quantitative data, or qualitative data, such as macrophage infiltration and NV, between the two groups (*p* > 0.05). Moreover, based on the cut-off values of LDL-C and APOB/A1 ratio for predicting NA, further comparison of the predictive value of LDL-C ≥ 2.51 mmol/L combined with APOB/A1 ≥ 0.87 compared with that of LDL-C ≥ 2.51 mmol/L for plaque morphology showed that the combined group had a higher proportion of NA and plaque erosion (*p* < 0.05), while similarities were present between the two groups in TCFA, intimal rupture, macrophage infiltration, NV, and thrombus (*p* > 0.05) (Fig. 3-B).

**Table 4:** Comparison of clinical data for different LDL-C levels

		Overall (n = 216)	LDL-C < 2.51 mmol/L (n = 123)	LDL-C ≥ 2.51 mmol/L (n = 93)	*p* value
**General information**					
	Age, year	64.00 (56.00–71.00)	64.00 (55.00–70.00)	65.00 (56.00–72.00)	0.181
	Male	172 (79.6)	100 (81.3)	72 (77.4)	0.483
	Current smoker	74 (34.3)	39 (31.7)	35 (37.6)	0.363
	Hypertension	128 (59.3)	52 (55.9)	76 (61.8)	0.384
	Diabetes mellitus	68 (31.5)	42 (34.1)	26 (28.0)	0.332
	LVEF, %	56.00 (49.25-61.00)	57.00 (51.00–61.00)	56.00 (44.00-59.50)	0.211
	Creatinine, µmol/L	84.00 (70.00-100.00)	85.00 (70.00-100.00)	84.00 (70.00-101.00)	0.871
	eGFR, mL/min/1.73 m^2^	103.27 (83.69-129.94)	101.77 (84.98-129.81)	104.20 (83.50-133.29)	0.687
	eGFR < 60, mL/min/1.73 m^2^	15 (6.9)	6 (4.9)	9 (9.7)	0.169
	Fasting blood glucose, mmol/L	5.75 (4.91–6.77)	5.64 (4.76–6.59)	5.83 (5.10–6.93)	0.190
	TG, mmol/L	1.69 (1.24–2.56)	1.58 (1.17–2.39)	1.87 (1.31–2.75)	0.044
	TC, mmol/L	3.88 (3.42–4.83)	3.57 (3.21–3.87)	4.87 (4.39–5.70)	< 0.001
	HDL-C, mmol/L	1.12 ± 0.24	1.13 ± 0.24	1.09 ± 0.25	0.239
	APOA1, g/L	1.20 (1.07–1.33)	1.18 (1.07–1.31)	1.23 (1.08–1.37)	0.157
	APOB, g/L	0.73 (0.60–0.90)	0.62 (0.55–0.71)	0.93 (0.81–1.08)	< 0.001
	APOB/A1	0.61 (0.49–0.77)	0.52 (0.44–0.61)	0.75 (0.63–0.96)	< 0.001
	Stent age, months	48.00 (25.25-84.00)	48.00 (24.00–84.00)	60.00 (25.50–91.50)	0.557
**Medication at restenosis**					
	Aspirin	176 (81.5)	105 (85.4)	71 (76.3)	0.091
	Statin	183 (84.7)	106 (86.2)	77 (82.8)	0.494
	ACEI/ARB	176 (81.5)	105 (85.4)	71 (76.3)	0.091
	β-blocker	179 (82.9)	103 (83.7)	76 (81.7)	0.697
	Insulin	12 (5.6)	8 (6.5)	4 (4.3)	0.484

Data are expressed as the median (interquartile range), mean ± SD, or n (%). ACEI: angiotensin-converting enzyme inhibitor; APO: apolipoprotein; ARB: angiotensin receptor blocker; eGFR, estimated glomerular filtration rate; HDL-C: high-density lipoprotein cholesterol; LDL-C: low-density lipoprotein cholesterol; LVEF: left ventricular ejection fraction; TC: total cholesterol; TG: triglyceride

**Table 5:** CAG and OCT findings for different LDL-C levels

			Overall (n = 216)	LDL-C < 2.51 mmol/L (n = 123)	LDL-C ≥ 2.51 mmol/L (n = 93)	*p* value
**CAG finding**						
	Lesion length, mm		12.05 (8.80-17.15)	11.50 (8.60–16.70)	12.60 (8.85–17.85)	0.154
	Multivessel disease		170 (78.7)	91 (74.0)	79 (84.9)	0.051
	Reference vessel diameter, mm		3.19 ± 0.41	3.19 ± 0.41	3.19 ± 0.41	0.924
	MLD, mm		1.16 ± 0.22	1.16 ± 0.21	1.15 ± 0.24	0.945
	Diameter stenosis, %		63.63 ± 6.00	63.53 ± 5.73	63.76 ± 6.37	0.784
	ISR location					0.922
		LAD	133 (61.6)	75 (61.0)	58 (62.4)	
		LCX	23 (10.6)	14 (11.4)	9 (9.7)	
		RCA	60 (27.8)	34 (27.6)	26 (28.0)	
	ISR pattern					0.395
		Focal	86 (39.8)	52 (42.3)	34 (36.6)	
		Diffuse	130 (60.2)	71 (57.7)	59 (63.4)	
	Previous stent type					
		First-generation DES	159 (73.6)	88 (71.5)	71 (76.3)	0.889
		New-generation DES	46 (21.3)	29 (23.6)	17 (18.3)	
		Unknown	11 (5.1)	6 (4.9)	5 (5.4)	
**OCT finding**						
	**Quantitative analysis**					
		MLA, mm^2^	1.68 ± 0.58	1.72 ± 0.57	1.62 ± 0.60	0.223
		Stent area (MLA site), mm^2^	6.58 ± 1.62	6.70 ± 1.68	6.43 ± 1.55	0.236
		Neointimal tissue area (MLA site), mm ^2^	4.90 ± 1.47	4.97 ± 1.52	4.81 ± 1.40	0.408
		Burden of neointimal tissue (MLA site), %	73.81 ± 8.73	73.53 ± 8.26	74.17 ± 9.36	0.594
	**Qualitative analysis**					
		NA	117 (54.2)	52 (42.3)	65 (69.9)	< 0.001
		TCFA	42 (19.4)	17 (13.8)	25 (26.9)	0.016
		Intimal rupture	40 (18.5)	16 (13.0)	24 (25.8)	0.017
		Plaque erosion	60 (27.8)	27 (22.0)	33 (35.5)	0.028
		Macrophage	42 (19.4)	22 (17.9)	20 (21.5)	0.506
		NV	96 (44.4)	54 (43.9)	42 (45.2)	0.854
		Thrombus	54 (25.0)	24 (19.8)	30 (32.3)	0.038

Data are expressed as the median (interquartile range), mean ± SD, or n (%). CAG: coronary angiography; DES: drug eluting stent; ISR: in-stent restenosis; LAD: left anterior descending artery; LCX: left circumflex artery; LDL-C: low-density lipoprotein cholesterol; MLA: minimum lumen area; MLD: minimal lumen diameter; NA: neoatherosclerosis; NV: neovascularization; OCT: optical coherence tomography; RCA: right coronary artery; TCFA: thin-cap fibroatheroma

**Fig. 4:**
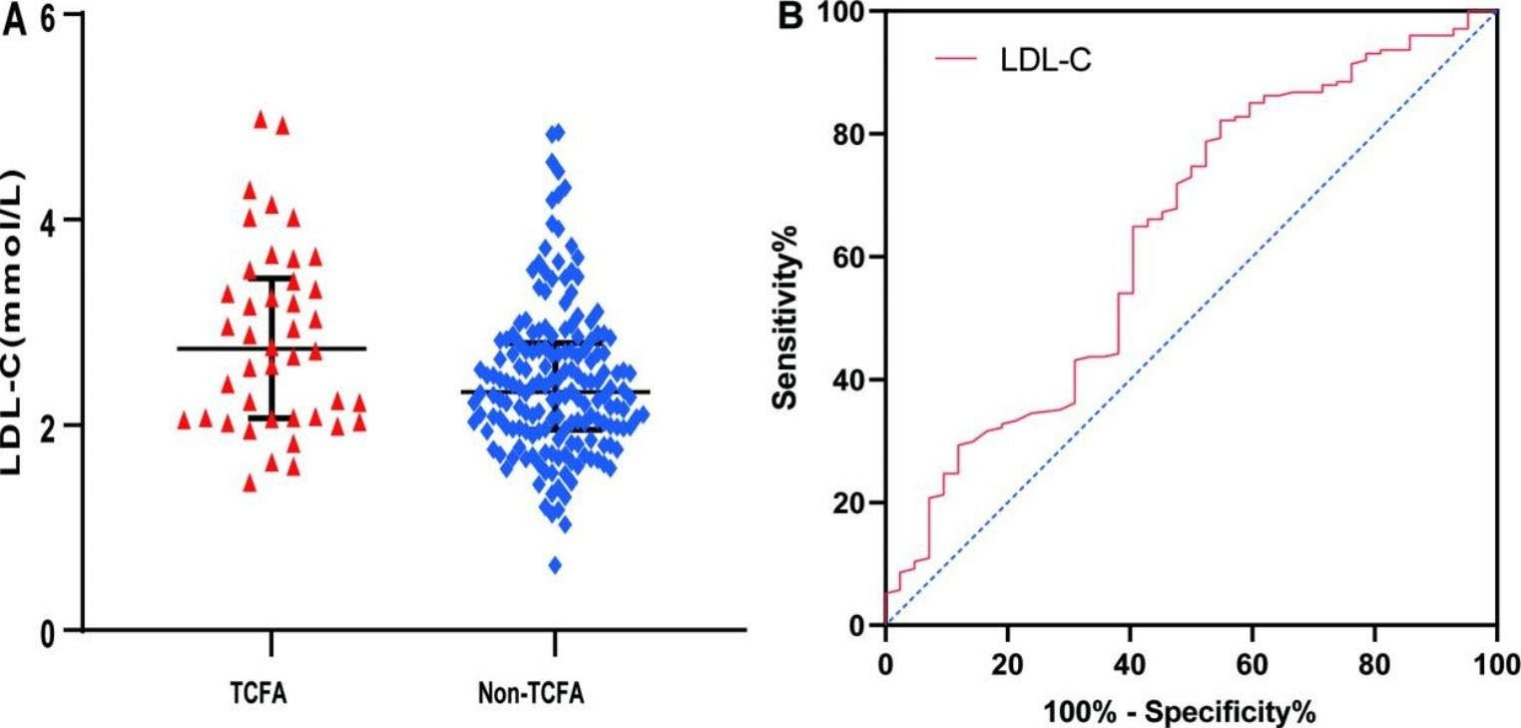
LDL-C level and ROC curve analysis for TCFA. LDL-C: low-density lipoprotein cholesterol; ROC: receiver operating characteristic; TCFA: thin-cap fibroatheroma

In addition, LDL-C levels in the TCFA group were significantly higher than those in the non-TCFA group (*p* = 0.006) (Fig. 4-A). Multivariate regression analysis showed that high LDL-C level was a risk factor for TCFA formation (odds ratio [OR], 1.916 [95% CI, 1.017–3.610]; *p* = 0.044) (Online Resources 5 and 6). To compare the predictive value of lipid types and stent placement time for TCFA, we screened statistically significant lipid variables by univariate regression analysis to further draw the ROC curve. The results showed that the AUCs for LDL-C, APOB/A1 ratio, TC, and stent age were 0.637 (95% CI, 0.540–0.734; *p* = 0.006) (Fig. 4-B), 0.616 (95% CI, 0.516–0.717; *p* = 0.019), 0.617 (95% CI, 0.518–0.716; *p* = 0.018), and 0.604 (95% CI, 0.513–0.700; *p* = 0.036), respectively (Online Resource 8).

**Fig. 5:**
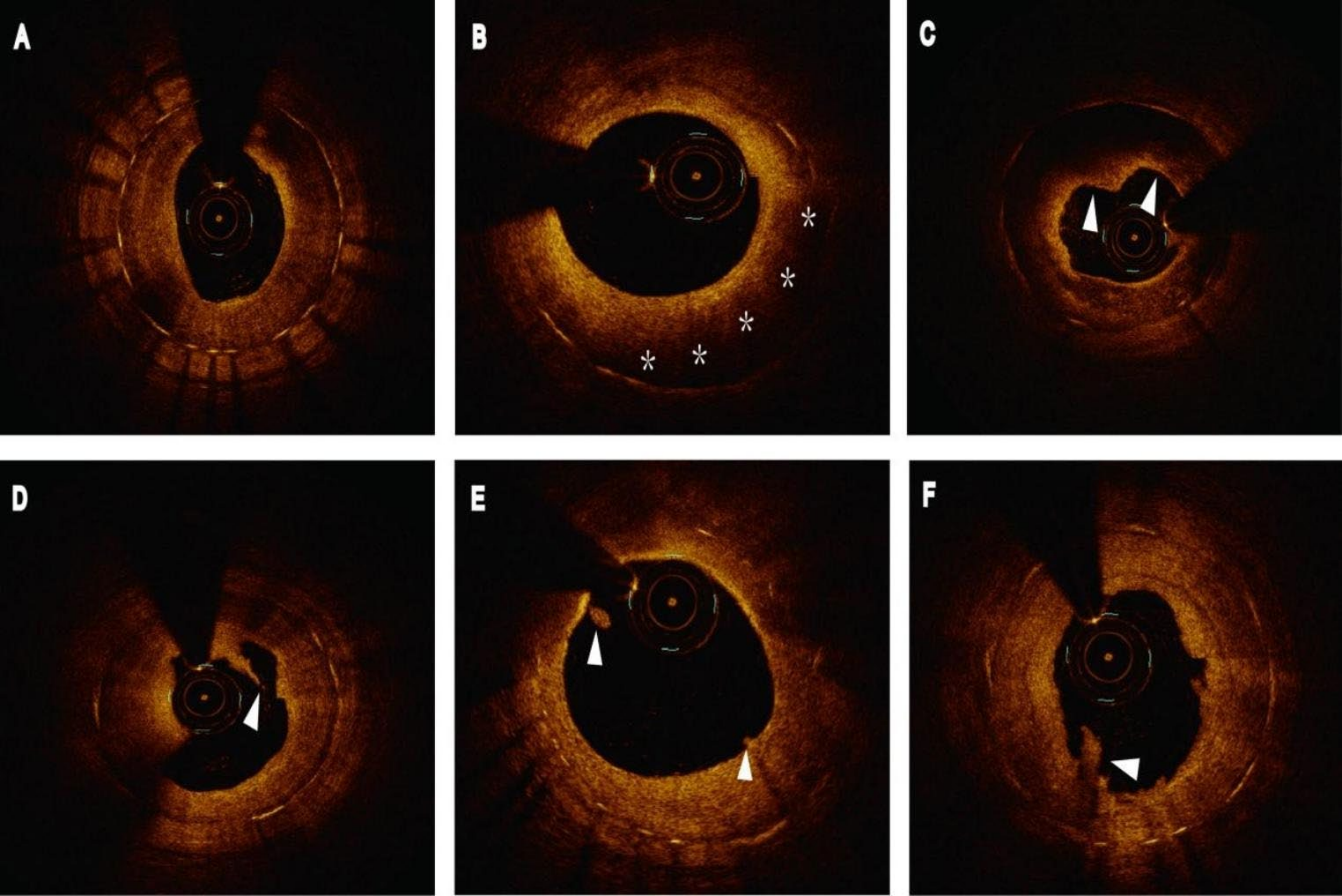
A case of late ISR with a high LDL-C level. OCT images showed NA and vulnerable plaque formation. (**A**) Heterogeneous neointima. (**B**) NA (asterisks). (**C**) Thin-cap fibroatheroma (white arrowheads). (**D**) Intimal disruption (arrowhead). (**E**) Plaque erosion (arrowheads). (**F**) Thrombus (arrowhead). ISR: in-stent restenosis; LDL-C: low-density lipoprotein cholesterol; NA, neoatherosclerosis; OCT: optical coherence tomography

A representative example of late ISR with a high LDL-C level is shown in Fig. 5. An 81-year-old woman with left anterior descending artery implanted with a DES for 34 months was followed-up with angiography due to ischemic symptoms. She had hypertension and no history of diabetes. LDL-C was 2.58 mmol/L at follow-up angiography. OCT images showed NA and vulnerable plaque formation.

### Reproducibility of qualitative OCT analysis

The kappa coefficients of inter- and intra-observer consistencies for NA and TCFA were 0.90/0.92 and 0.87/0.89, respectively.

## Discussion

Increasing evidence has revealed that the incidence of NA is time-dependent, and the role of the lipid profile in NA formation is still controversial. In particular, no systematic studies have explored the predictors of NA incidence and plaque vulnerability in patients with late ISR. In light of this, this study systematically analyzed the predictors of NA incidence and plaque vulnerability in patients with late ISR, and the results indicated the following: (1) the incidence of NA in patients with late ISR was 54.2%, and the proportion of patients with ACS in the NA group was higher than that in the non-NA group during follow-up CAG; (2) stent age and lipid profiles were related to the formation of NA and TCFA, in which the AUC of LDL-C level was higher than that of stent age and other lipid types; (3) the incidences of TCFA, intima rupture, plaque erosion, and thrombosis in the LDL-C ≥ 2.51 mmol/L group were significantly higher than those in the LDL-C < 2.51 mmol/L group. Moreover, the incidences of NA and plaque erosion in the LDL-C ≥ 2.51 mmol/L combined with APOB/A1 ≥ 0.87 group were significantly higher than those in the LDL-C ≥ 2.51 mmol/L group.

### The incidence and clinical presentation of NA in patients with late ISR

NA has been a significant contributor to stent failure, especially in the late stages, and its formation can induce MACEs [1, 4, 9]. Notably, NA formation cannot be avoided, even with the use of new-generation DES, and its incidence gradually increases with prolonged stent implantation time [2]. Yonetsu et al. [22] previously reported that when the stent implantation time was > 48 months, the incidence of NA was > 70% for both bare-metal stents (BMS) and DES. A study by Chen et al. [18] revealed that the incidence of NA was as high as 75% when stents were implanted for > 7 years. Nakamura et al. [1] included 313 ISR lesions (20.4% BMS, 23.3% first-generation DES, and 53.7% new-generation DES) with a median stent age of 732 days and NA incidence of 47.0%. In our study, the incidence of NA was also high (54.2%), and the proportion of patients with ACS at CAG follow-up in the NA group was significantly higher than that in the non-NA group, which further revealed that NA formation may contribute to MACEs.

### Predictors of late ISR with NA and its correlation with LDL-C level

Despite the theoretical association between NA and late DES failure, little is known about the possible risk factors for late ISR with NA formation. Previous studies have revealed that the occurrence of NA is related to factors such as stent age, stent type, current smoking status, CKD, and the use of ACEI/ARB [1, 5, 6]. Meanwhile, a growing number studies have revealed that the occurrence and development of NA are time-dependent [18, 22, 23] which shows that NA formation will be more common with extended stent implantation time. In addition, some studies have shown that LDL-C levels of > 70 mg/dL are independent predictors of NA [2]. Masaru et al. [9] found that a high LDL-C level was the main risk factor for NA progression. A recent OCT study of early ISR combined with NA also found that poor LDL-C control after percutaneous coronary intervention (PCI) was the main cause of early NA [10]. However, Sakai et al. [12] reported that in patients receiving statin therapy after PCI, triacylglycerol-rich lipoprotein cholesterol and APOB were involved in early NA formation, but LDL-C was not. Therefore, whether LDL-C plays a role in NA formation in patients with late ISR remains unknown. In the present study, univariate regression analysis revealed that blood creatinine, eGFR, stent age, TG, TC, LDL-C, APOB, and APOB/A1 ratio were associated with NA formation. Multivariate regression analysis revealed that LDL-C, TG, and the time from PCI to ISR were associated with NA formation. These findings indicate that LDL-C levels play a key role in late ISR with NA formation, even with the use of statins. To avoid interactions between lipid types in the multivariate analysis, confounding factors were further adjusted for lipid types with statistical differences in the univariate regression analysis to evaluate their roles in NA formation. The results indicated that lipid profiles play an important role in NA formation. TG, TC, LDL-C, APOB, and APOB/A1 ratio were still related to NA formation after multifactor correction. In addition, stent age was still associated with NA formation after correction for different lipid types. Notably, the AUC of the LDL-C level for predicting NA was larger than that of stent age and other lipid types, suggesting that intensive lipid-lowering therapy is still important for patients with late ISR, and the control of LDL-C levels is still an important target of lipid-lowering therapy.

In this study, the type of stent, CKD, and current smoking status were not associated with NA formation, which may be related to the special population included in this study, i.e., patients with late ISR. This further suggests that the mechanism of NA in patients with late ISR may differ from that in patients with early ISR, which needs to be confirmed by large-sample prospective studies.

### The correlation between LDL-C level and plaque vulnerability in patients with late ISR

Vulnerable plaques are closely related to the occurrence of MACEs. The FOURIER and ODYSSEY OUTCOMES studies have shown that the combination of proprotein convertase subtilisin/kexin type 9 (PCSK9) inhibitors with statin therapy could effectively reduce LDL-C levels and the risk of primary endpoint events [24, 25]. However, the risk factors for plaque vulnerability in patients with late ISR have not been reported; thus, whether intensive lipid-lowering therapy is also suitable for patients with late ISR in a real clinical environment is not clear. Therefore, the early identification of risk factors for plaque vulnerability in patients with late ISR and timely and appropriate interventions are particularly important to improve prognosis. In addition, the COMBINE OCT-FFR trial showed that TCFA, one of the important characteristics of vulnerable plaques, was the strongest predictor of MACEs [21]. Recently, the PACMAN-AMI trial revealed that intensive lipid-lowering therapy increased fibrous cap thickness [26]. Another study showed that intensive lipid-lowering therapy promoted an increase in fibrous cap thickness and regression of lipid-rich plaques in patients with ACS, which was associated with a greater reduction in LDL-C [8]. Therefore, this study explored the association between LDL-C levels and TCFA, with the results indicating that LDL-C levels in the TCFA group were significantly higher than those in the non-TCFA group. Logistic regression models revealed that LDL-C level was an important risk factor for TCFA formation (*p <* 0.01), and its AUC was higher than that for stent age and other lipid types. Moreover, subgroup analysis according to the cut-off value of LDL-C showed that the incidences of NA, TCFA, intimal rupture, plaque erosion, and thrombosis in the LDL-C ≥ 2.51 mmol/L group were higher than those in the LDL-C < 2.51 mmol/L group. Deng et al. [27] showed that the APOB/A1 ratio was correlated with the vulnerability of coronary plaques in patients with atherosclerotic cardiovascular disease. Our study also showed that the APOB/A1 ratio was associated with NA and TCFA formation, but its AUC was smaller than that of LDL-C level. Further analysis suggested that the LDL-C ≥ 2.51 mmol/L combined with APOB/A1 ≥ 0.87 group had a higher proportion of NA and plaque erosion compared with the LDL-C ≥ 2.51 mmol/L group. Therefore, the increased APOB/A1 ratio is not only related to the vulnerability of the plaque in situ, but also to the vulnerability of the plaque in patients with late ISR. This provides a new perspective for controlling blood lipid levels in patients with late ISR, and the significance of an increased APOB/A1 ratio cannot be ignored.

### Limitations

First, this was a single-center, retrospective study with a relatively small sample size. Second, OCT data at the time of stent implantation were lacking. Third, the study only included patients with late ISR who had available OCT data and complete clinical data and excluded patients who lacked OCT imaging, had incomplete clinical data, or did not meet the inclusion criteria, which may have led to a potential selection bias. Therefore, the data derived from this study may not be representative of a broader patient population. Fourth, previous studies [28] have indicated that macrophage infiltration plays a significant role in plaque vulnerability. However, this study did not extensively investigate the relationship between intra-plaque inflammation and the formation of NA. In future research, we are committed to further exploring the association between intra-plaque inflammation and the development of NA. Fifth, although OCT is currently the preferred intravascular imaging method for diagnosing NA and identifying vulnerable plaques, it has limitations and may not accurately assess qualitative neointimal characteristics.

## Conclusion

A high LDL-C level is an independent predictor of NA and TCFA formation in patients with late ISR, and its predictive value may exceed stent implantation time and other lipid types such as APOB and APOB/A1 ratio. Therefore, actively controlling LDL-C levels remains an important strategy for preventing and reducing the incidence of NA and plaque vulnerability in patients with late ISR, which is expected to be further confirmed by prospective studies.

### Electronic supplementary material

Below is the link to the electronic supplementary material.

Supplementary Material 1
