# Supplementary material for: Association of LDL-C level with neoatherosclerosis and plaque vulnerability in patients with late restenosis: an optical coherence tomography study
Source: Int J Cardiovasc Imaging. 2023 Oct 7;39(12):2609–19. doi: 10.1007/s10554-023-02956-1 (PMC10691981; doi:10.1007/s10554-023-02956-1)
Supplement: Supplementary file 1 — Supplementary Material 1 [file 10554_2023_2956_MOESM1_ESM.docx]

*Original Article*

**Association of LDL-C level with neoatherosclerosis and plaque vulnerability in patients with late restenosis: An optical coherence tomography study**

Zhijiang Liu^1,a^, Chancui Deng^1,a^, Ranzun Zhao^1,a^, Guanxue Xu^2^, Zhixun Bai^1^, Zhenglong Wang^1^, Wei Zhang^1^, Yi Ma^1^, Xingwei Hu^1^, Caide Jin^1^, Panke Chen^1^, Shuai Ma^1^, and Bei Shi^1^

^1^ Department of Cardiology, Affiliated Hospital of Zunyi Medical University, Zunyi, China

^2^ Department of Cardiology, the Fifth Affiliated Hospital of Zunyi Medical University, Zhuhai, China

^a^ Zhijiang Liu, Chancui Deng, and Ranzun Zhao contributed equally to this paper and share first authorship.

Corresponding Author:

Bei Shi

Department of Cardiology, Affiliated Hospital of Zunyi Medical University, Zunyi 563000, China

E-mail: [shibei2147@163.com](mailto:shibei2147@163.com)

**Online Resource 1**

1. **Data Collection and Angiographic Analysis**

The demographic and clinical characteristics of the patients were collected by two trained technicians, who were blinded to the study objectives, by reviewing the patients’ hospital records. Blood samples were obtained from patients after 10 h of fasting on the morning of the coronary angiography (CAG), and specific indicators included blood glucose, estimated glomerular filtration rate (eGFR), and lipid profile. The eGFR was calculated using the Kidney Disease Study Group equation. Coronary angiography was analyzed offline using quantitative coronary analysis (QCA) software (Artis VC21C, Siemens AG, Berlin and Munich, Germany). The angiographic classification of in-stent restenosis (ISR) was based on Mehran's classification [[1](#Reference1)], while QCA analysis was conducted by two experienced coronary interventionalists who were blinded to the clinical data of the patients.

1. **Optical Coherence Tomography Image Acquisition**

Optical coherence tomography (OCT) images were acquired using a frequency-domain OCT, the C7XR/ILUMIEN/ILUMIEN OPTIS Intracavitary Imaging System (St. Jude Medical, St. Paul, MN, USA). The imaging process was based on a previous expert consensus [[2](#Reference2)]. Briefly, the OCT imaging catheter was extended into the distal end of the target lesion at a distance of 5 mm, and the image was acquired at the appropriate scanning speed. During the OCT image acquisition, a contrast agent was administered to clear the blood vessels. All the OCT images were digitally stored for subsequent offline analysis.

1. **Definitions**

Morphometric analyses were performed using the standard definition of the cross-sectional area measurements reported previously [[2-4](#Reference2)]. The neointima was defined as the tissue between the margin of the vascular lumen and the inner edge of the stent trabeculae. The neointimal burden was calculated as follows: (neointimal area × 100) / stent area. Homogeneous neointima was defined as the area within the intima with homogeneous signal-rich bands and without local changes or attenuation. Heterogeneous neointima was defined as the region of the inner membrane where local optical properties were altered and various backscattering patterns were observed. Intimal disruption was defined as disruption of the continuous middle segment of the intimal lumen. Plaque erosion was defined as an intact fibrous cap without plaque rupture accompanied by thrombosis and an identifiable plaque structure under the thrombosis, or an intact fibrous cap, absence of thrombosis in the culprit lesion, irregular luminal surface or presence of thrombosis at the lesion, indistinct plaque structure at the thrombus, and absence of superficial lipids or calcification proximal or distal to the thrombus [[5](#Reference5)]. Macrophages were characterized by specular or banded structures with high reflection and strong attenuation on OCT images, and radiated shadows are often formed behind the specular region with a high signal [[2](#Reference2)]. Neovascularization (NV) was defined as the presence of a circular or tubular structure spanning the neointimal tissue in at least three consecutive frames [[6](#Reference6)]. A thrombus was defined as an irregularly shaped mass that protruded into the lumen and was not coherent with the wall.

1. **Statistical Analysis**

Data analysis was performed using SPSS software (version 20.0; IBM, Armonk, NY, USA). Categorical data are presented as numbers (percentages), and differences between groups were compared using the chi-square test or Fisher's exact test. The distribution of continuous data was assessed by the Kolmogorov–Smirnov test; normally distributed continuous data are presented as means ± standard deviations (SDs). An independent sample t-test was used to compare the differences between the two groups; skewed continuous data are presented as medians (interquartile ranges), and the Mann–Whitney U test was used to compare the differences between the two groups. Univariate and multivariate logistic regression analyses were used to evaluate variables known or suspected to be associated with neoatherosclerosis (NA) and thin-cap fibroatheroma (TCFA). Among them, the univariate retrospective analysis selected age, sex, current smoking status, hypertension, diabetes mellitus, stent age, use of angiotensin-converting enzyme inhibitor/angiotensin receptor blocker, use of first-generation drug-eluting stent, left ventricular ejection fraction, fasting blood glucose, serum creatinine, eGFR, eGFR <60 mL/min/1.73 m^2^, triglyceride, total cholesterol, low-density lipoprotein cholesterol (LDL-C), apolipoprotein A1 (APOA1), APOB, and APOB/A1 ratio as variables with *p*-values of <0.01, which were included in the multivariate logistic analysis. Second, multiple logistic regression analysis models of lipid profiles (univariate logistic regression analyses, *p*<0.05) and stent age were constructed to predict NA, and a regression model of the LDL-C level was constructed to predict TCFA. The results were expressed as odds ratios and 95% confidence intervals. The predicted NA and TCFA values of the LDL-C level were further detected using receiver operating characteristic curve analysis, and the area under the curve, 95% confidence interval, and *p*-values were calculated. Results were considered statistically significant if a 2-sided *p-*value was <0.05. Furthermore, the inter- and intra-observer reproducibility of the imaging analysis was evaluated using the kappa coefficient.

**References**

1. Mehran R, Dangas G, Abizaid AS, et al (1999) Patterns of in-stent restenosis : Angiographic classification and implications for long-term clinical outcome. Circulation. 100(18):1872-8. https://doi: 10.1161/01.cir.100.18.1872
2. Co-Chair G, Regar CE, Co-Chair T, et al (2012) Consensus Standards for Acquisition, Measurement, and Reporting of Intravascular Optical Coherence Tomography Studies. Journal of the American College of Cardiology 59(12):1058-1072. J Am Coll Cardiol 59(12):1058-72.

https://doi: 10.1016/j.jacc.2011.09.079

1. Araki M, Park S-J, Dauerman HL, et al (2022) Optical coherence tomography in coronary atherosclerosis assessment and intervention. Nat Rev Cardiol 19(10):684-703. https://doi: 10.1038/s41569-022-00687-9
2. Takano M, Yamamoto M, Inami S, et al (2009) Appearance of Lipid-Laden Intima and Neovascularization After Implantation of Bare-Metal Stents. J Am Coll Cardiol 55(1):26-32. https://doi.org/10.1016/j.jacc.2009.08.032
3. Jia H, Abtahian F, Aguirre AD, et al (2013) In Vivo Diagnosis of Plaque Erosion and Calcified Nodule in Patients With Acute Coronary Syndrome by Intravascular Optical Coherence Tomography. J Am Coll Cardiol 62(19):1748-1758. https://doi.org/10.1016/j.jacc.2013.05.071
4. Shibuya M, Fujii K, Hao H, et al (2015) Characterization of In-Stent Neointima Using Optical Coherence Tomography in the Late Phase After Bare-Metal Stent Implantation--An Ex Vivo Validation Study. Circ J 79(10):2224-2230.

https://doi.org/10.1253/circj.CJ-15-0585

1. **Supplementary Tables**

**Online Resource 2 Logistic regression analysis for NA**

| Variables | Univariate model | | |  | Multivariate model | | |
| --- | --- | --- | --- | --- | --- | --- | --- |
|  | OR | 95% CI | *p*-value |  | OR | 95% CI | *p*-value |
| Age, year | 1.012 | (0.986-1.038) | 0.374 |  |  |  |  |
| Male | 0.981 | (0.505-1.907) | 0.955 |  |  |  |  |
| Current smoker | 0.853 | 0.484-1.502 | 0.581 |  |  |  |  |
| Hypertension | 1.198 | 0.694-2.068 | 0.517 |  |  |  |  |
| Diabetes mellitus | 1.277 | 0.718-2.271 | 0.405 |  |  |  |  |
| LVEF, % | 0.977 | 0.949-1.005 | 0.112 |  |  |  |  |
| Creatinine, μmol/L | 1.011 | 1.000-1.022 | 0.047 |  | 0.997 | 0.978-1.015 | 0.720 |
| eGFR, mL/min/1.73 m^2^ | 0.992 | 0.984-1.000 | 0.047 |  | 0.990 | 0.975-1.006 | 0.209 |
| eGFR <60, mL/min/1.73 m^2^ | 0.273 | 0.075-0.998 | 0.050 |  | 0.571 | 0.099-3.303 | 0.531 |
| Fasting blood glucose, mmol/L | 0.989 | 0.880-1.111 | 0.849 |  |  |  |  |
| ACEI/ARB | 0.504 | 0.244-1.040 | 0.064 |  | 0.584 | 0.259-1.318 | 0.195 |
| First-generation DES | 1.197 | 0.653-2.194 | 0.561 |  |  |  |  |
| Stent age, months | 1.007 | 1.000-1.013 | 0.041 |  | 1.008 | 1.001-1.015 | 0.024 |
| TG, mmol/L | 1.330 | 1.052-1.681 | 0.017 |  | 1.315 | 1.021-1.695 | 0.034 |
| TC, mmol/L | 1.298 | 1.036-1.625 | 0.023 |  | 0.965 | 0.666-1.399 | 0.852 |
| HDL-C, mmol/L | 0.377 | 0.122-1.160 | 0.089 |  | 0.546 | 0.148-2.017 | 0.364 |
| LDL-C, mmol/L | 2.263 | 1.509-3.396 | <0.001 |  | 2.068 | 1.062-4.026 | 0.033 |
| APOA1, g/L | 0.886 | 0.269-2.921 | 0.843 |  |  |  |  |
| APOB, g/L | 6.080 | 1.837-20.125 | 0.003 |  | 0.493 | 0.038-6.403 | 0.589 |
| APOB/A1 | 5.847 | 1.769-19.329 | 0.004 |  | 3.337 | 0.560-19.877 | 0.186 |

ACEI: angiotensin-converting enzyme inhibitor; APO: apolipoprotein; ARB: angiotensin receptor blocker; CI: confidence interval; DES: drug eluting stent; eGFR: estimated glomerular filtration rate; HDL-C: high-density lipoprotein cholesterol; LDL-C: low-density lipoprotein cholesterol; LVEF: left ventricular ejection fraction; NA: neoatherosclerosis; OR: odds ratio; TC: total cholesterol; TG: triglyceride.

**Online Resource 3 Multiple logistic regression analysis models for evaluating the association between lipid profiles and** **risk of NA**

| Variables | Model 1 | | |  | Model 2 | | |  | Model 3 | | |
| --- | --- | --- | --- | --- | --- | --- | --- | --- | --- | --- | --- |
|  | OR | 95% CI | *p*-value |  | OR | 95% CI | *p*-value |  | OR | 95% CI | *p*-value |
| TG | 1.391 | 1.087-1.780 | 0.009 |  | 1.482 | 1.140-1.926 | 0.003 |  | 1.517 | 1.151-1.998 | 0.003 |
| TC | 1.300 | 1.038-1.628 | 0.022 |  | 1.292 | 1.031-1.621 | 0.026 |  | 1.269 | 1.004-1.602 | 0.046 |
| LDL-C | 2.258 | 1.505-3.389 | <0.001 |  | 2.163 | 1.429-3.272 | <0.001 |  | 2.157 | 1.409-3.302 | <0.001 |
| APOB | 6.279 | 1.894-20.815 | 0.003 |  | 6.010 | 1.770-20.463 | 0.004 |  | 6.116 | 1.739-21.502 | 0.005 |
| APOB/A1 | 6.963 | 2.035-23.822 | 0.002 |  | 7.145 | 2.048-24.924 | 0.002 |  | 7.322 | 2.036-26.333 | 0.002 |

Model 1: adjusted for age and sex.

Model 2: adjusted for confounders in Model 1 plus current smoking, hypertension, diabetes mellitus, stent age, ACEI/ARB use, and first-generation DES.

Model 3: adjusted for confounders in Model 2 plus LVEF, fasting blood glucose, creatinine, eGFR, and eGFR <60mL/min/1.73 m^2^.

ACEI: angiotensin-converting enzyme inhibitor; APO: apolipoprotein; ARB: angiotensin receptor blocker; CI: confidence interval; DES: drug-eluting stent; eGFR, estimated glomerular filtration rate; LDL-C: low-density lipoprotein cholesterol; LVEF: left ventricular ejection fraction; NA: neoatherosclerosis; OR: odds ratio; TC: total cholesterol; TG: triglyceride.

**Online Resource 4 Multiple logistic regression analysis models to evaluate the association between stent age and risk of NA**

| Models | OR | 95% CI | *p*-value |
| --- | --- | --- | --- |
| Model 1 | 1.008 | 1.001-1.015 | 0.027 |
| Model 2 | 1.009 | 1.001-1.016 | 0.020 |
| Model 3 | 1.008 | 1.001-1.015 | 0.028 |
| Model 4 | 1.008 | 1.001-1.015 | 0.032 |
| Model 5 | 1.008 | 1.001-1.015 | 0.031 |
| Model 6 | 1.008 | 1.001-1.015 | 0.027 |

Model 1: adjusted for age, sex, current smoking, hypertension, diabetes mellitus, stent age, ACEI/ARB, first-generation DES, LVEF, fasting blood glucose, creatinine, eGFR, and eGFR <60mL/min/1.73 m^2^.

Model 2: adjusted for confounders in Model 1 plus TG.

Model 3: adjusted for confounders in Model 1 plus TC.

Model 4: adjusted for confounders in Model 1 plus LDL-C.

Model 5: adjusted for confounders in Model 1 plus APOB.

Model 6: adjusted for confounders in Model 1 plus APOB/A1.

ACEI: angiotensin-converting enzyme inhibitor; APO: apolipoprotein; ARB: angiotensin receptor blocker; CI: confidence interval; DES: drug-eluting stent; eGFR: estimated glomerular filtration rate; LDL-C: low-density lipoprotein cholesterol; LVEF: left ventricular ejection fraction; NA: neoatherosclerosis; OR: odds ratio; TC: total cholesterol; TG: triglyceride.

**Online Resource 5 Logistic regression analysis for TCFA**

| Variables | Univariate model | | |  | Multivariate model | | |
| --- | --- | --- | --- | --- | --- | --- | --- |
|  | OR | 95% CI | *p*-value |  | OR | 95% CI | *p*-value |
| Age, year | 1.011 | (0.979-1.044) | 0.512 |  |  |  |  |
| Male | 0.923 | (0.402-2.107) | 0.850 |  |  |  |  |
| Current smoker | 0.923 | 0.456-1.869 | 0.825 |  |  |  |  |
| Hypertension | 0.676 | 0.333-1.372 | 0.278 |  |  |  |  |
| Diabetes mellitus | 1.031 | 0.498-2.135 | 0.934 |  |  |  |  |
| LVEF, % | 0.975 | 0.943-1.009 | 0.149 |  |  |  |  |
| Creatinine, μmol/L | 1.020 | 1.008-1.033 | 0.001 |  | 1.014 | 0.976-1.053 | 0.474 |
| eGFR, mL/min/1.73 m^2^ | 0.984 | 0.973-0.995 | 0.005 |  | 1.002 | 0.974-1.030 | 0.912 |
| eGFR <60, mL/min/1.73 m^2^ | 0.131 | 0.044-0.393 | <0.001 |  | 0.393 | 0.062-2.514 | 0.324 |
| Fasting blood glucose, mmol/L | 0.943 | 0.803-1.808 | 0.474 |  |  |  |  |
| ACEI/ARB | 0.667 | 0.296-0.667 | 0.328 |  |  |  |  |
| First-generation DES | 1.184 | 0.540-2.596 | 0.673 |  |  |  |  |
| Stent age, months | 1.008 | 1.001-1.015 | 0.035 |  | 1.008 | 1.000-1.016 | 0.045 |
| TG, mmol/L | 0.978 | 0.749-1.728 | 0.872 |  |  |  |  |
| TC, mmol/L | 1.302 | 1.027-1.652 | 0.029 |  | 1.202 | 0.833-1.733 | 0.326 |
| HDL-C, mmol/L | 1.016 | 0.261-3.963 | 0.981 |  |  |  |  |
| LDL-C, mmol/L | 1.893 | 1.252-2.861 | 0.002 |  | 1.916 | 1.017-3.610 | 0.044 |
| APOA1, g/L | 0.727 | 0.165-3.203 | 0.673 |  |  |  |  |
| APOB, g/L | 2.964 | 0.887-9.899 | 0.077 |  | 0.100 | 0.007-1.543 | 0.099 |
| APOB/A1 | 3.243 | 1.079-9.743 | 0.036 |  | 5.306 | 0.944-29.811 | 0.058 |

ACEI: angiotensin-converting enzyme inhibitor; APO: apolipoprotein; ARB: angiotensin receptor blocker; CI: confidence interval; DES: drug-eluting stent; eGFR: estimated glomerular filtration rate; HDL-C: high-density lipoprotein cholesterol; LDL-C: low-density lipoprotein cholesterol; LVEF: left ventricular ejection fraction; OR: odds ratio; TC: total cholesterol; TCFA: thin-cap fibroatheroma; TG: triglyceride.

**Online Resource 6** **Logistic regression analysis models to evaluate the association between LDL-C levels and the risk of TCFA**

| Models | OR | 95% CI | *p*-value |
| --- | --- | --- | --- |
| Model 1 | 1.894 | 1.252-2.867 | 0.003 |
| Model 2 | 1.870 | 1.222-2.861 | 0.004 |
| Model 3 | 1.802 | 1.157-2.807 | 0.009 |

Model 1: adjusted for age and sex.

Model 2: adjusted for confounders in Model 1 plus current smoking, hypertension, diabetes mellitus, stent age, ACEI/ARB use, and first-generation DES.

Model 3: adjusted for confounders in Model 2 plus LVEF, fasting blood glucose, creatinine, eGFR, and eGFR <60mL/min/1.73 m^2^.

ACEI: angiotensin-converting enzyme inhibitor; ARB: angiotensin receptor blocker; CI: confidence interval; DES: drug-eluting stent; eGFR, estimated glomerular filtration rate; LVEF: left ventricular ejection fraction; OR: odds ratio; TCFA: thin-cap fibroatheroma.

1. **Supplementary Figures**

**Online Resource 7 Receiver operating characteristic curve analyses to predict neoatherosclerosis in patients with late in-stent restenosis**


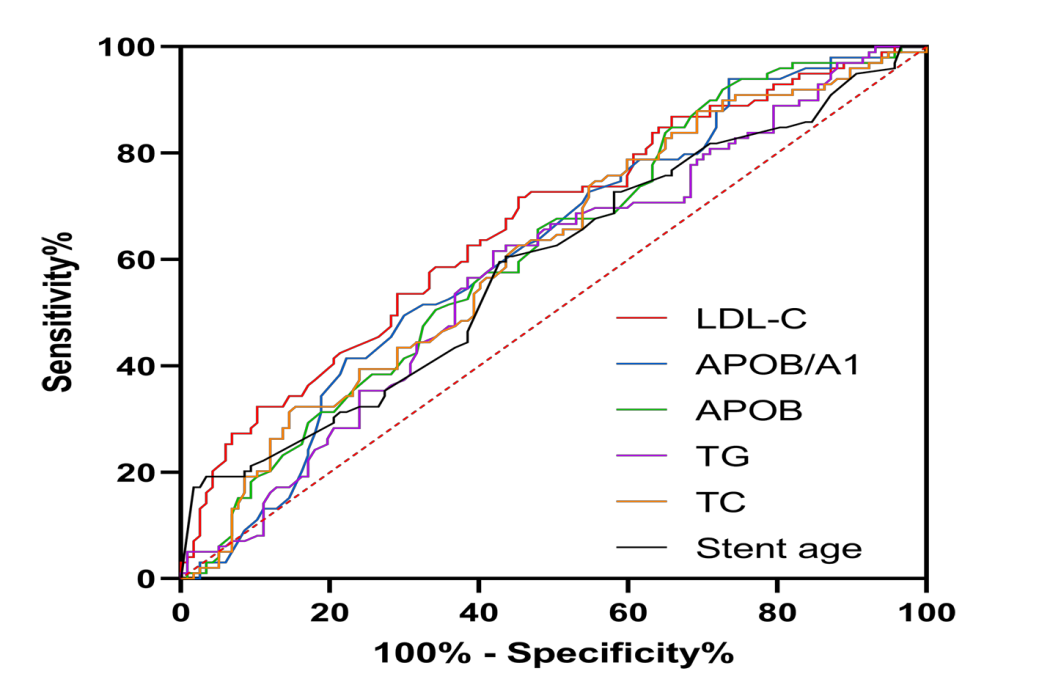


APO: apolipoprotein; LDL-C: low-density lipoprotein cholesterol; TC: total cholesterol; TG: triglyceride.

**Online Resource 8 Receiver operating characteristic curve analyses to predict thin-cap fibroatheroma in patients with late in-stent restenosis**

**
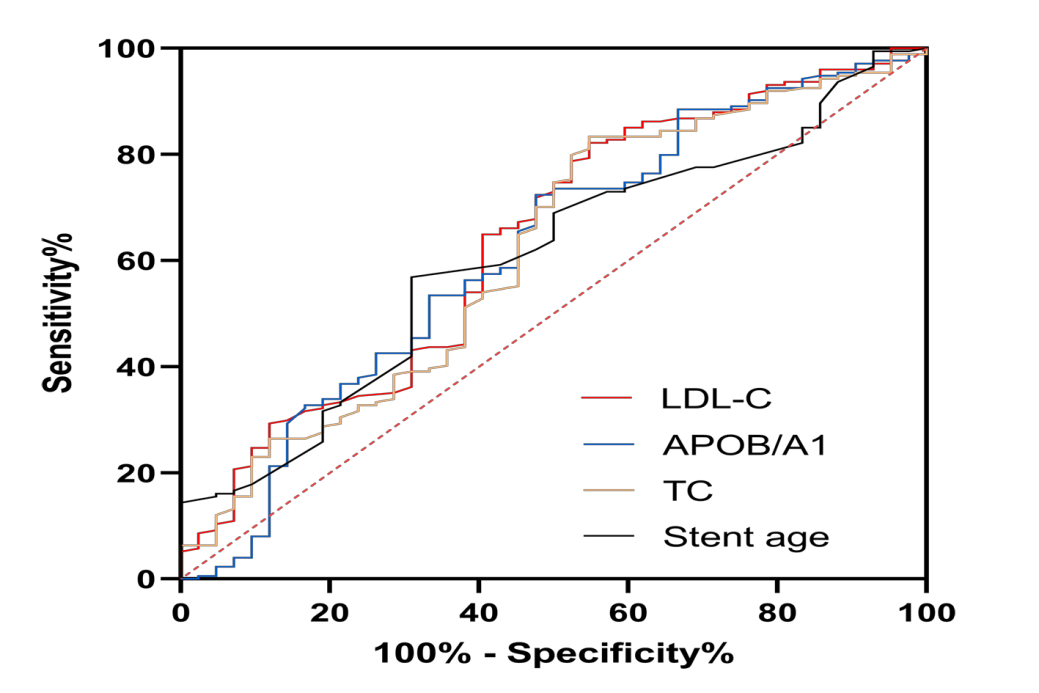
**

APO: apolipoprotein; LDL-C: low-density lipoprotein cholesterol; TC: total cholesterol.
